# Muscle atrophy induced by overexpression of ALAS2 is related to muscle mitochondrial dysfunction

**DOI:** 10.1186/s13395-021-00263-8

**Published:** 2021-03-30

**Authors:** Yahui Peng, Jihong Li, Dixian Luo, Shuai Zhang, Sijia Li, Dayong Wang, Xidi Wang, Zhujun Zhang, Xue Wang, Changhui Sun, Xu Gao, Yang Hui, Rongzhang He

**Affiliations:** 1 Department of Biochemistry and Molecular Biology, Harbin Medical University, Harbin, 150086 China; 2 Institute of Translational Medicine, National and Local Joint Engineering Laboratory of High-through Molecular Diagnostic Technology, the First People’s Hospital of Chenzhou, The First Affiliated Hospital of Xiangnan University, Chenzhou, 423000 China; 3 Heilongjiang Academy of Medical Sciences, Harbin, 150086 China; 4 Key Laboratory of Preservation of Human Genetic Resources and Disease Control in China (Harbin Medical University), Ministry of Education, Beijing, 150086 China; 5 Department of Clinical Pharmacology, Xiangya Hospital, Central South University, Changsha, 410078 China

**Keywords:** Muscle atrophy, Mitochondrial dysfunction, Transgenic mice, Delta-aminolevulinate synthase 2

## Abstract

**Background:**

ALAS2 (delta-aminolevulinate synthase 2) is one of the two isoenzymes catalyzing the synthesis of delta-aminolevulinic acid (ALA), which is the first precursor of heme synthesis. ALAS2-overexpressing transgenic mice (Tg mice) showed syndrome of porphyria, a series of diseases related to the heme anabolism deficiency. Tg mice showed an obvious decrease in muscle size. Muscle atrophy results from a decrease in protein synthesis and an increase in protein degradation, which ultimately leads to a decrease in myofiber size due to loss of contractile proteins, organelles, nuclei, and cytoplasm.

**Methods:**

The forelimb muscle grip strength of age-matched ALAS-2 transgenic mice (Tg mice) and wild-type mice (WT mice) were measured with an automated grip strength meter. The activities of serum LDH and CK-MB were measured by Modular DPP. The histology of skeletal muscle (quadriceps femoris and gastrocnemius) was observed by hematoxylin and eosin (HE) staining, immunohistochemistry, and transmission electron microscope. Real-time PCR was used to detect mtDNA content and UCP3 mRNA expression. Evans blue dye staining was used to detect the membrane damage of the muscle fiber. Single skeletal muscle fiber diameter was measured by single-fiber analyses. Muscle adenosine triphosphate (ATP) levels were detected by a luminometric assay with an ATP assay kit.

**Results:**

Compared with WT mice, the strength of forelimb muscle and mass of gastrocnemius were decreased in Tg mice. The activities of serum CK-MB and LDH, the number of central nuclei fibers, and Evans blue positive fibers were more than those in WT mice, while the diameter of single fibers was smaller, which were associated with suppressed expression levels of MHC, myoD1, dystrophin, atrogin1, and MuRF1. Re-expression of eMyHC was only showed in the quadriceps of Tg mice, but not in WT mice. Muscle mitochondria in Tg mice showed dysfunction with descented ATP production and mtDNA content, downregulated UCP3 mRNA expression, and swelling of mitochondria.

**Conclusion:**

ALAS2 overexpressing-transgenic mice (Tg mice) showed muscle dystrophy, which was associated with decreased atrogin-1 and MuRF-1, and closely related to mitochondrial dysfunction.

**Supplementary Information:**

The online version contains supplementary material available at 10.1186/s13395-021-00263-8.

## Background

The heme biosynthetic pathway begins from delta-aminolevulinic acid synthase (ALAS) catalyzing the condensation of glycine and succinyl-CoA to delta-aminolevulinic acid (ALA) in the mitochondria [1]. ALAS is coded by two genes: ALAS1 and ALAS2 [2]. ALAS1 is ubiquitously expressed in all cells, and the negative feedback is regulated by the heme pool [3, 4]; however, ALAS2 is specifically expressed only in erythroid cells [2, 5] and is not inhibited by heme [6]. Bechara reported that reactive oxygen species (ROS) is formed by the metal-catalyzed aerobic oxidation of ALA at abnormally high levels [6, 7]; mitochondria are the main source of ROS and are also the primary target of oxidant-induced damage. ALA, as a putative endogenous source of ROS, induces mitochondrial swelling and transmembrane potential collapse [7], and ALA-treated rats under swimming training experienced fatigue earlier [8]. Defective mitochondrial function has been shown to cause muscle weakness [9]. The loss of mitochondria has also been shown to result in muscle wasting [10]. Notably, studies have reported abnormalities in the mitochondria during sarcopenia, muscle wasting, associated with chronic illness (cachexia), and disuse atrophy [11, 12].

The mitochondria produce adenosine triphosphate (ATP) as a source of chemical energy, and skeletal muscle contains abundant mitochondria. Increased mitochondrial ROS production can promote disuse muscle atrophy by increasing proteolysis and depressing protein synthesis, and ROS can contribute to mitochondrial damage and impaired the ability to produce ATP, which results in energy stress [10]. Theoretically, mitochondrial damage could decrease the level of cellular energy available for protein synthesis, and energy stress could promote proteolysis via the AMPK-FoxO3 axis [10].

Disorder of the heme biosynthesis pathway could induce porphyria. Each enzymatic alteration of the heme biosynthesis system can cause a specific porphyria [13]. The clinical manifestations include acute neurovisceral attacks, skin lesions, and muscle atrophy, which are associated with the accumulation of porphyrin precursors (5-aminolevulinic acid, porphobilinogen) and porphyrins. Muscle weakness due to porphyria can progress and lead to tetraplegia, with respiratory and bulbar paralysis [13]. ALAS2-overexpressing transgenic mice (Tg mice) showed obvious muscle atrophy. Jasmin Barman-Aksözen et al. found a significant increase in the amount of ALAS2 mRNA and protein among patients with erythropoietic protoporphyria (EPP) [14]. Additionally, four recurrent gain-of-function mutations in the catalytic domain of the ALAS2 enzyme resulting in an increased ALAS2 activity have been described as being responsible for X-linked protoporphyria (XLPP) [15]. To-Figueras et al. presented convincing evidence that ALAS2 acts as a modifier gene in patients with congenital erythropoietic porphyria [16]. In this study, we reported that ALAS2 overexpressing-transgenic mice (Tg mice) showed muscle atrophy, which was associated with decreased atrogin-1 and MuRF-1, and closely related to mitochondrial dysfunction.

## Materials and methods

### Animals

ALAS2 transgenic mice were generated by the standard pronuclear injection technique using C57BL/6 mice. Briefly, mouse ALAS2 cDNA was cloned into the pCAGGS plasmid with the chicken β-actin promoter, which drove the mouse ALAS2 cDNA expression, and terminated with the poly (A) signal. All animals were identified by analysis of tail DNA by PCR. Sequences of primers were as follows (5′−3′): forward primer was GCCTTCTTCTTTTTCCTACAGCTC; reverse primer was GCACAATCTTGCTCTTCCTGTCTTGG. Both the ALAS2 transgenic mice and wild type mice were housed in a temperature-controlled room (22 °C) with a 12-h light to 12-h dark cycle. Unless otherwise noted, 6- to 12-month-old male mice were used in the experiments. All procedures were approved by the Institutional Animal Care and Use Committee of Harbin Medical University.

### Measurement of forelimb muscle grip strength

The forelimb muscle grip strength was measured by an automated grip strength meter (Jinan, China) as described previously [17]. Briefly, mice were lifted by their tails and made to hold a horizontal bar with their forelimbs. Next, they were pulled slowly backwards until they could no longer hold the grip. The maximal force was recorded during consecutive attempts (at least 20 attempts per mouse), and the average was set as the result.

### Biochemical assays

Serum samples were separated from whole blood by centrifugation at 1,000×g for 10 minutes after the blood was allowed to clot at room temperature for 30 minutes. The activities of serum lactate dehydrogenase (LDH), creatine kinase (CK), and creatine kinase-MB (CK-MB) were measured by Modular DPP (Roche).

### Processing of tissues for histology

The gastrocnemius and/or quadriceps muscles were fixed with 4% formalin for at least 36 h. The tissues were embedded in paraffin and cut into 4 μm thick sections in the transverse myofilament direction. Then, the sections were stained with hematoxylin and eosin (HE), and the images were visualized and captured with the Olympus Bx51 microscope.

### Immunohistochemistry

The sections of paraffin-embedded muscle tissue were deparaffinized in xylene and rehydrated in ethyl alcohol. Then, the sections were blocked with 1% hydrogen peroxide (H_2_O_2_) in distilled water for 10 min, and the non-specific sites were blocked with bovine serum albumin (BSA, DAKO) for 20 min at room temperature. For detecting eMyHC, heat-induced antigen retrieval was performed (Tris/EDTA buffer, pH 8, DAKO) prior to staining the muscle samples. The sections were then incubated overnight at 4 °C with primary antibody of anti-eMyHC (clone BF-45, mouse, 1:400). The BF-45 monoclonal antibody was obtained from DSHB at the University of Iowa in USA. After thorough washing in PBS, the sections were incubated with biotin-conjugated secondary antibodies (DAKO) at 37 °C for 20 min. We used a standard peroxidase-based method with DAB (DAKO) to detect the antibody. The sections were dehydrated with ethyl alcohol and coverslipped with mounting medium. The stained sections were imaged using an Olympus BX51 microscope.

### Evans blue assay

Evans blue assay was performed as described previously [18]. Evans blue dye (10 mg/ml) was dissolved in phosphate buffered saline (PBS). Then, it was filtered sterilely by a 0.2-μm pore filter. The Evans blue dye was intraperitoneally injected into the mouse (0.1 ml/10 g body weight). The mice were killed 24 h after injection. The quadriceps muscle of these killed mice was prepared and observed under the Olympus Bx51microscope.

### Single-fiber analyses

Single fibers were isolated and fiber size was measured as described previously [19]. The quadriceps muscles were fixed with 4% paraformaldehyde (PFA) for more than 2 days. Dissected small bundles of fibers were incubated in 40% NaOH for 2-3 h and vigorously shaken. Isolated myofibers were washed in PBS and stained with 10 μM DAPI. Images of 40-60 single fibers per animal were captured with the Olympus Bx51 microscope, and fiber diameter was measured by Olympus Element software.

### Muscle ATP level

For the muscle ATP level, we used a luminometric assay with ATP Assay kit (Beyotime) according to the manufacturer’s instructions.

### Real-time PCR

The muscles were harvested from ALAS2 transgenic and wild-type (WT) mice and were frozen immediately in liquid nitrogen, and then they were stored frozen at – 80 °C. For RNA isolation, the tissue was homogenized in Trizol reagent (Invitrogen) and total RNA was prepared according to the manufacturer’s protocol. The RNA was reverse transcribed into cDNA by High-Capacity cDNA Reverse Transcription kit (Applied Biosystems). Real-time PCR (RT-PCR) analyses were performed by the ABI 7500 real-time PCR system (Applied Biosystems). The glyceraldehyde-3-phosphate dehydrogenase (GAPDH) expression was used to normalize the expression levels. The relative expression values gained were used to calculate fold change. The primer sequences are listed in Table 1 (5′−3′).

**Table 1:**
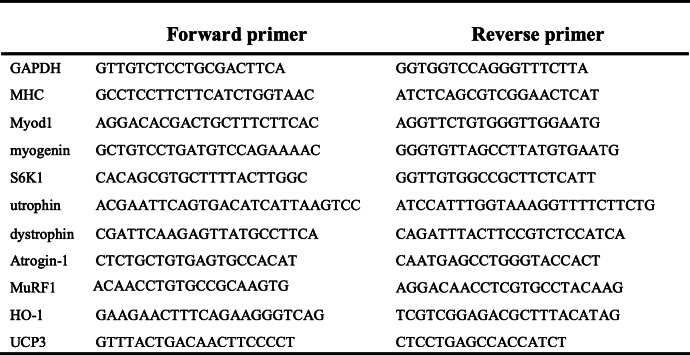
Primers table

### Mitochondrial DNA (mtDNA) content assay

The muscles were digested and the DNA was isolated using the DNeasy Blood and Tissue kit (QIAGEN). The mtDNA content was quantified by qRT-PCR using a SYBR Green-based detection system by the ABI 7500 real-time PCR system (Applied Biosystems) according to the manufacturer’s protocol in a similar way as the previous description [20]. The qRT-PCR primer sequences of mtDNA and nucleus DNA were as follows (5′-3′), mtDNA: Forward primer was AAGTCGTAACAAGGTAAGCA, and Reverse primer was ATATTTGTGTAGGGCTAGGG; Nuc.DNA: Forward primer was GGGTATATTTTTGATACCTTCAATGAGTTA, and Reverse primer was TCTGAAACAGTAGGTAGAGACCAAAGC.

### Transmission electron microscopy (TEM)

The muscle blocks were prepared and soaked immediately in 2.5% glutaraldehyde. After 6-8 h at 4 °C, they were cut into 1mm thick coronal slices. Next, the samples were rinsed with PBS (0.1 M) before being post-fixed by osmium tetroxide for 1-2 h. The muscle blocks were dehydrated through a graded series of alcohol and acetone. Subsequently, we used epoxy resin for embedding prior to slicing of the ultra-thin sections. Then, double staining by uranyl acetate and lead citrate was performed. Finally, the images were acquired by a transmission electron microscope (JEM-1220, JEOL Ltd, Tokyo, Japan).

### Western blotting

About 20 mg muscle tissue was lysed in RIPA Lysis Buffer (Beyotime) for 10 minutes on ice. RIPA Lysis Buffer is configured with 20 mM Tris PH7.5, 150 mM NaCl, 1% Triton X-100, 2.5 mM sodium pyrophosphate, 1 mM EDTA, 1%Na_3_VO_4_, 0.5 μg/ml leupeptin, and 1 mM phenyl methane sulfonyl fluoride (PMSF). The lysate homogenate was centrifuged at 12,000×*g* at 4 °C for 5 min. The protein concentration was measured with the DC Protein Assay kit (Bio-Rad Laboratories). Protein samples were boiled for 10 min in the presence of 4× Loading Dye. Equal amounts of total proteins (25 μg) were loaded on a 12% SDS-polyacrylamide gel for electrophoresis followed by a transfer to PVDF membranes (Millipore) at 70 V for 1 h. The membranes were blocked with 5% non-fat powdered milk in PBS (10 mM, pH 7.4) for 1 h at 4 °C. The blot was incubated with the primary antibody (GAPDH, 1:2000, Cell Signaling Technology, HO-1, Santa Cruz Biotechnology) overnight at 4 °C. The membrane was washed three times by PBST, followed by incubation with the appropriate secondary antibody. The signal was detected by an Enzymatic Chemiluminescence (ECL) kit (Applygen).

### Statistics

All quantitative data are expressed as means ± SD. Statistical analysis was performed using either Student’s *t* test (two groups) or one-way analysis of variance (more than two groups), followed by Bonferroni post hoc test. Differences were considered significant at *P* < 0.05.

## Results

### Reduction of forelimb muscle grip strength in ALAS2 transgenic mice

**Fig. 1:**
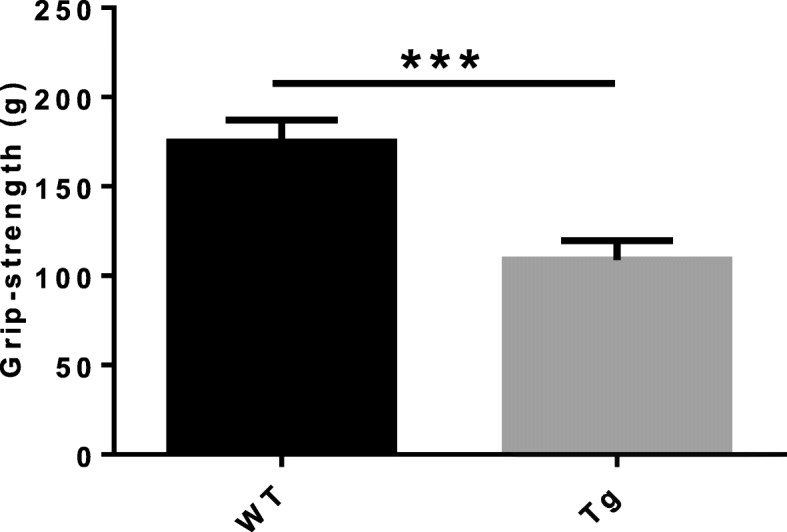
Reduced forelimb muscle grip strength in ALAS2 transgenic mice. Grip strength in ALAS2 transgenic mice (*n* = 9) and WT mice (*n* = 8). Values are means ± SD. *P < 0.05;**P < 0.01;***P < 0.001

As muscle weakness is a clinical manifestation of porphyria [18, 21, 22], we measured the forelimb muscle grip strength by the automated grip strength meter. Interestingly, we found that ALAS2 transgenic (Tg) mice had reduced forelimb muscle strength compared with the age-matched WT littermates (Fig. 1). The data strongly suggest that muscle weakness is present in ALAS2 transgenic (Tg) mice.

### Loss of muscle mass in ALAS2 transgenic mice

**Fig. 2:**
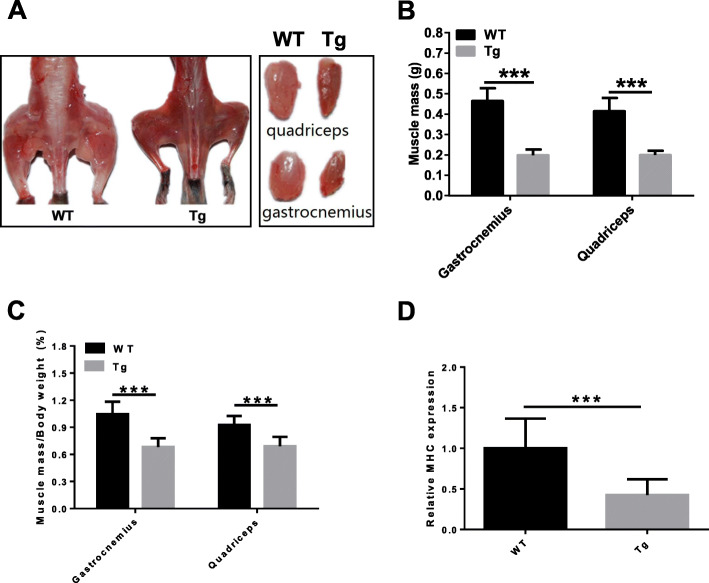
Loss of muscle mass in the ALAS2 transgenic mice. **a** Left panel: gross morphology of skinned hindlimb muscles of ALAS2 transgenic mice and WT mice. Right panel: comparison of individual muscles. **b** Comparison of changes in wet weight of individual muscles mass (*n* = 6–7). **c** Comparison of in wet weight of individual muscles mass normalized to body weight (*n* = 6–7). **d** The MHC mRNA expression (*n* = 6–7). Values are means ± SD.*P < 0.05; **P < 0.01; ***P < 0.001

Tg mice were smaller and thinner than the age-matched WT littermates (data not shown). On visual analysis, overall loss of hindlimb muscle mass was clearly evident in Tg mice (Fig. 2a). The wet weight of the quadriceps femoris was approximately half of that in the age-matched WT littermates, and this finding was similar to that in the gastrocnemius muscle (Fig. 2b). As Tg mice were smaller and thinner, we normalize the wet weight of the quadriceps femoris mass by the body weight. The results showed that the muscle mass percentage in Tg mice was lower than that in WT mice (Fig. 2c). The myosin heavy chain (MHC) mRNA expression was also measured. We found the MHC mRNA level in Tg mice was decreased compared with that in WT mice (Fig. 2d). The data indicated muscle mass loss in Tg mice.

### Muscle atrophy in ALAS2 transgenic mice

**Fig. 3:**
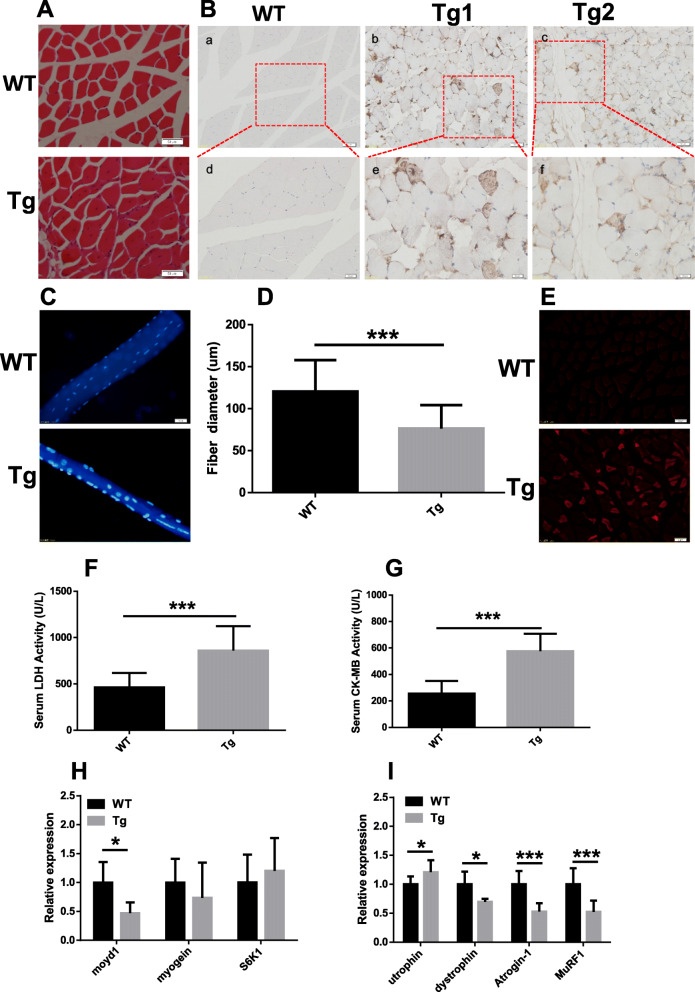
Muscle atrophy in the ALAS2 transgenic mice. **a** Quadriceps cross section from hematoxylin and eosin stain. (bar, 50 μm). **b** Expression of eMyHC in WT and ALAS2 transgenic mice (Tg) muscle. (Bar of a-c is 50 μm, and bar of d-e 20 μm). **c** Single-fiber size comparison of WT and ALAS2 transgenic mice quadriceps muscle. **d** Quantification of average fiber diameter across single fibers in quadriceps muscle from WT (*n* = 4) and ALAS2 transgenic mice (*n* = 4). **e** Evans blue dye was intraperitoneal injected and mice sacked 24 h later. Transverse cross-sections of quadriceps muscle were observed with OLYMPUS BX51 equipped with green activation filters. Evans blue-positive fibers are denoted by red staining. Bar, 50 μm. **f** Serum LDH levels of WT (*n* = 7) and ALAS2 transgenic mice (*n* = 6). **g** Serum CK-MB levels of WT (*n* = 7) and ALAS2 transgenic mice (*n* = 6). **h** The relative mRNA expression of myod1, myogenin, and S6K1 (*n* = 5–8). **i** The relative mRNA expression of utrophin, dystrophin, Atrogin-1, and MuRF1. Values are means ± SD.*P < 0.05; **P < 0.01; ***P < 0.001

To determine the cause of loss of muscle mass, HE-stained transverse sections of the quadriceps femoris from age-matched Tg mice and WT mice were compared. A high number of muscle fibers with centrally located nuclei was found in Tg mice, a primary pathological sign of muscular dystrophy [23], but not in WT mice (Fig. 3a). Moreover, to detect the regeneration of the quadriceps in Tg mice, re-expression of eMyHC were compared by immunostaining for quadriceps cross section from age-matched Tg mice and WT mice at 6 months old. Re-expression of eMyHC was only showed in the quadriceps of Tg mice, but not in WT mice (Fig. 3b), indicating that the central nucleation determined in Tg mice muscles resulted from muscle regeneration [24, 25]. With respect to single-fiber analyses [19, 26], the average diameter of single fibers isolated from the muscle of Tg mice was smaller than that isolated from the muscle of WT mice (Fig. 3c, d). In addition, to examine leakage into the muscle fiber, Evans blue assay was employed [18, 27]. The fluorescent dye accumulated in the interior of dystrophied muscle fibers in Tg mice (Fig. 3e). We showed that the myocyte membrane was damaged in dystrophied muscle of Tg mice. Moreover, we detected the activities of serum CK, CK-MB, and LDH, and we found the elevation of the activity of serum CK-MB and LDH in Tg mice (Fig. 3f, g). We analyzed the expression of the genes, and we found that the expression levels of MyoD1, dystrophin, Atrogin-1, and MuRF1 were decreased, but the expression level of utrophin was increased. There was no difference in myogenin and S6K1 in Tg mice compared with WT mice (Fig. 3h, i). Albertyn CH et al. also described that acute intermittent porphyria presenting as progressive muscular atrophy in a 23-year-old black South African man [12].

### Mitochondrial dysfunction in the muscle of ALAS2 transgenic mice

**Fig. 4:**
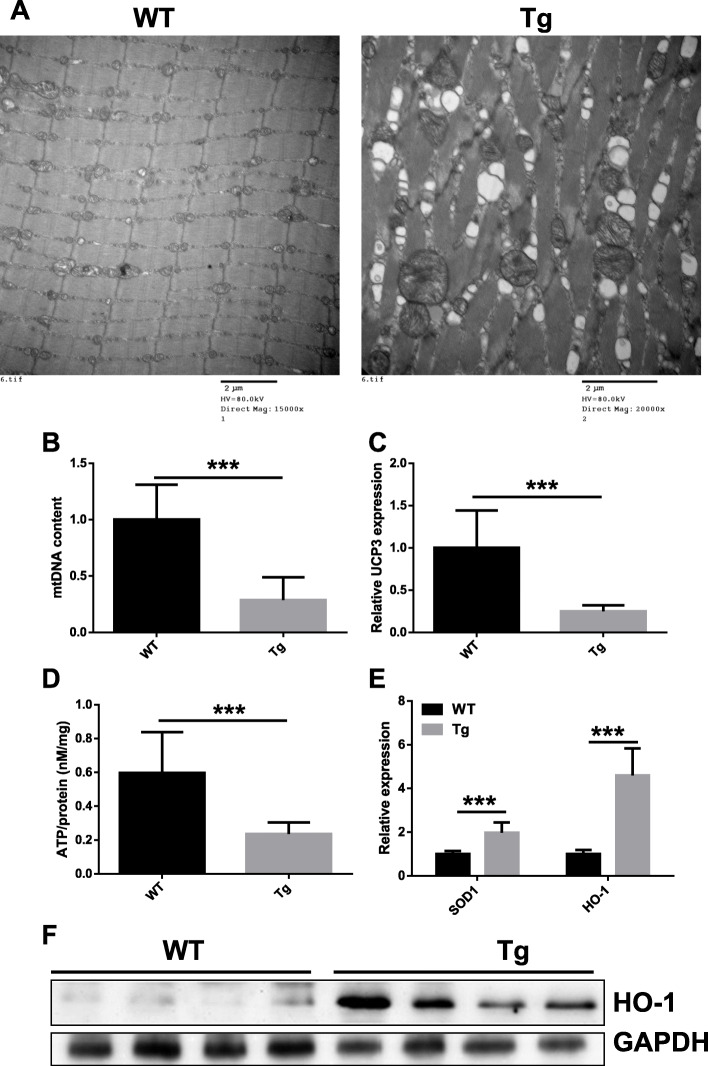
Mitochondrial dysfunction in the ALAS2 transgenic mice muscle. **a** TEM images of gastrocnemius of WT and ALAS2 transgenic mice. Bars, 2 μm. **b** mtDNA content in gastrocnemius of WT (*n* = 8) and ALAS2 transgenic mice (*n* = 7). **c** UCP3 expression in quadriceps of WT (*n* = 8) and ALAS2 transgenic mice (*n* = 7). **d** ATP production in gastrocnemius of WT (*n* = 8) and ALAS2 transgenic mice (*n* = 8). **e** The SOD1 and HO-1 mRNA expression(*n* = 5–8). **f** The HO-1 protein expression (*n* = 4). Values are means ± SD. *P < 0.05; **P < 0.01; ***P < 0.001

To test whether muscle dystrophy in Tg mice was affected by mitochondrial damage, we examined the ultrastructure of muscle fibers using a transmission electron microscope. Mitochondrial swelling was found in muscles of Tg mice (Fig. 4a, right), but not in muscles of WT mice **(**Fig. 4a, left). Then we compared the mtDNA content, uncoupling protein 3 (UCP-3) mRNA expression, and ATP production in the hind limb muscles of Tg mice and age-matched WT mice. The mtDNA content quantified by qRT-PCR [20] was significantly reduced in the gastrocnemius muscle of Tg mice (Fig. 4b). The muscle UCP-3 mRNA expression was decreased in Tg mice (Fig. 4c). ATP production in the gastrocnemius muscle of Tg mice was decreased to 21% of the level in age-matched WT mice (Fig. 4d). Interestingly, increased expression levels of SOD1 mRNA, HO-1 mRNA, and HO-1 protein showed that SOD1 and HO-1 were induced in the muscle of Tg mice (Fig. 4e, f). Above all, mitochondrial dysfunction and loss were present in the muscle of Tg mice. It was reported that mitochondrial energetic failure played an important role in the expression of acute intermittent porphyria (AIP) [28].

## Discussion

ALAS2-overexpressing Tg mice were developed to investigate the mechanism of porphyria. The expression of ALAS2 was increased in Tg mice [29]. Tg mice showed obvious muscular atrophy, which is also a clinical characteristic of porphyria. Therefore, we explored the mechanism of muscular atrophy in Tg mice. Firstly, we found that Tg mice experienced a decrease in muscle mass and grip strength of the forelimb muscles. Secondly, increased activities of serum CK-MB and LDH, increased central nuclear fiber and Evans blue positive fiber and decreased single-fiber diameter confirmed muscle atrophy in Tg mice. In addition, Re-expression of eMyHC was only showed in the quadriceps of Tg mice, but not in WT mice, indicating that the central nucleation determined in Tg mice muscles resulted from muscle regeneration. Furthermore, the expression levels of MyoD1, S6K1 (anabolic factor), atrogin1, and MuRF1 (catabolic factor) were determined. Finally, muscle mitochondrial dysfunction in ALAS2 Tg mice was detected based on mitochondrial swelling, decline in ATP production and mtDNA content, and downregulation of UCP3 mRNA expression.

We found that the muscle grip strength of forelimbs of Tg mice was decreased. Since muscle mass determines the skeletal muscle strength [30], the loss of muscle strength in Tg mice may be caused by the loss of muscle mass. The diameter of single fibers of Tg mice was smaller than that of WT mice, and thinner fiber indicated less muscle mass. MHC is an important part of the sarcomere [31]. We found that the mRNA level of MHC in Tg mice was decreased, which meant that the loss of muscle mass may be caused by the decrease in MHC content. In addition to the decreased muscle mass of atrophic muscles, a large number of muscle fibers with centrally located nucleus were observed in Tg mice, which is a sign of muscle fiber regeneration [32, 33]. We also found that the re-expression of eMyHC was only showed in the quadriceps of Tg mice, but not in WT mice, confirming that the central nucleation determined in Tg mice muscles resulted from muscle regeneration. The Evans blue dye could enter into the myocyte through the damaged cytomembrane and get accumulated in the myocyte, and thus, Evans blue dye was used to identify damaged skeletal myofibers [27, 34, 35]. Accumulation of Evans blue dye in the myocyte of Tg mice suggested that the myocyte membrane was damaged in Tg mice. Also, increased activities of serum LDH and CK-MB indicated that the muscular membrane of Tg mice was damaged [36–38]. The expression of MyoD1 was decreased in Tg mice compared with WT mice. Since muscle regeneration has been reported to be delayed in MyoD (−/−) mice [39], decreased MyoD1 might cause a disturbance in the regeneration in Tg mice.

The expression of utrophin was increased and that of dystrophin was decreased in Tg mice compared with WT mice, which was similar to that in other dystrophy reports [33, 40]. MAFbx and MuRF1 belong to the ubiquitin proteasome pathway, which plays a critical role in the intracellular protein degradation of skeletal muscle [41]. Upregulation of atrophy-related genes atrogin-1 (MAFbX) and MuRF1 in skeletal muscle atrophy has been reported previously [42, 43]. However, atrogin-1 and MuRF1 were downregulated in aging-related loss of skeletal muscle [44] and in mTOR-mice [33], and here, we also found that atrogin-1 and MuRF1 levels were decreased in Tg mice. Inhibition of MuRF1 is sufficient to maintain the MHC [44]. However, MHC in Tg mice was decreased, which indicated that the loss of muscle mass in Tg mice was not related to activation of the ubiquitin proteasome pathway. A previous study showed that chronic spinal cord-injured patients with severe atrophy of the quadriceps muscles showed a reduction in atrogin-1 and MuRF1, which suggested an internal mechanism aimed at reducing the further loss of muscle proteins [45, 46]. The reduction of atrogin-1 and MuRF1 in Tg mice may also be a protective attempt to reduce further muscle wasting in muscle atrophy.

Mitochondrial dysfunction is a hallmark trait that occurs during prolonged muscle inactivity in both animals and humans. Mitochondrial fission and remodeling contribute to muscle atrophy [47]. Increased superoxide in vivo accelerates age-associated muscle atrophy through mitochondrial dysfunction [19]. Mitochondria play an important role in muscle atrophy [19, 47, 48]. It has been reported that ALA-generated oxidant promotes dysfunction and swelling of the isolated rat liver mitochondria [7]. Similarly, mitochondrial swelling and mitochondrial cristae reduction were shown in muscles of Tg mice. As Tg mice have high expression of ALAS2 [29] and accumulation of ALA in the muscles (data not shown), mitochondrial damage in the muscle of Tg mice is most likely to be induced by ALA. There is an increase of SOD-1 in the brain, muscle, and liver of chronic ALA-treated rats [8], and thus, we speculate that SOD-1 and HO-1 are induced by ALA in Tg mice. Increasing of  anti-oxidant enzymes in Tg mice indicated oxidant existence. As excessive free radicals accelerate muscle proteolysis [12, 49], the pro-oxidizing nature of ALA [50] may lead to the loss of muscle mass. Previous studies have indicated that exercise induced up-regulation of UCP-3 and downregulation of UCP-3 would damage the muscles [51, 52]. Decreased mtDNA content and UCP-3 expression suggested that the mitochondrial loss in Tg mice was correlated with mitochondrial damage. A decrease in the ATP production was observed in Tg mice, which was probably induced by mitochondrial damage and loss. The pCAGGS expression vector can drive EGFP expression in all tissues, except erythrocytes and hair in mice, particularly higher in the muscle [53]. Also, Tg mice have ubiquitous overexpression of ALAS-2 in all tissues and higher expression of ALAS-2 in the muscle. Moreover, the accumulation of ALA is much higher in Tg mice than in WT mice. Because ALA is synthesized in the mitochondria and ALA is a putative endogenous source of ROS [7], ALA might damage the mitochondria of muscle in Tg mice.

Porphyrias are a group of eight metabolic disorders of the heme biosynthesis pathway [7]. Every porphyria is caused by abnormal function of a separate enzymatic step, resulting in a specific accumulation of heme precursors, including ALA, PBG, and porphyrins. In some cases, muscle atrophy was present in porphyria; however, the underlying mechanism is still unknown. ALAS2-overexpressing Tg mice also show accumulation of ALA, thus it may be a new model of porphyria. In the future, we will further verify whether ALAS2-overexpressing Tg mice can be used as a porphyria model, and we will use this model to investigate the relationship of mitochondrial dysfunction and porphyria-related muscle weakness.

## Conclusion

Muscle weakness in ALAS2-overexpressing mice is related to muscle mitochondrial dysfunction induced by the accumulation of ALA.

## Supplementary Information

**Additional file 1 **: **FigS. 1** Overexpresion ALAS-2 in in mouse myoblasts (C2C12). A, The relative mRNA expression of ALAS-2. B, The relative mRNA expression of myod1, myogein and S6K1. C, The relative mRNA expression of utrophin, dystrophin, Atrogin-1 and MuRF1. Values are means ± SD.*P<0.05;**P<0.01;***P<0.001.

## Data Availability

All the data and material could be traced from the paper or can be requested from the corresponding author.

## Abbreviations

ALAS2: Delta-aminolevulinate synthase 2
ALA: Delta-aminolevulinic acid
Tg mice: Transgenic mice
WT mice: Wild-type mice
ATP: Adenosine triphosphate
HE: Hematoxylin and eosin
ROS: Reactive oxygen species
EPP: Erythropoietic protoporphyria
XLPP: X-linked protoporphyria
mtDNA: Mitochondrial DNA
LDH: Lactate dehydrogenase
CK: Creatine kinase
CK-MB: Creatine kinase-MB
GAPDH: Glyceraldehyde-3-phosphate dehydrogenase
TEM: Transmission electron microscopy
