# Supplementary material for: Muscle atrophy induced by overexpression of ALAS2 is related to muscle mitochondrial dysfunction
Source: Skelet Muscle. 2021 Mar 30;11:9. doi: 10.1186/s13395-021-00263-8 (PMC8008657; doi:10.1186/s13395-021-00263-8)
Supplement: Supplementary file 1 — Additional file 1 : FigS. 1 Overexpresion ALAS-2 in in mouse myoblasts (C2C12). A, The relative mRNA expression of ALAS-2. B, The relative mRNA expression of myod1, myogein and S6K1. C, The relative mRNA expression of utrophin, dystrophin, Atrogin-1 and MuRF1. Values are means ± SD.*P<0.05;**P<0.01;***P<0.001. [file 13395_2021_263_MOESM1_ESM.pptx]

## Slide 1
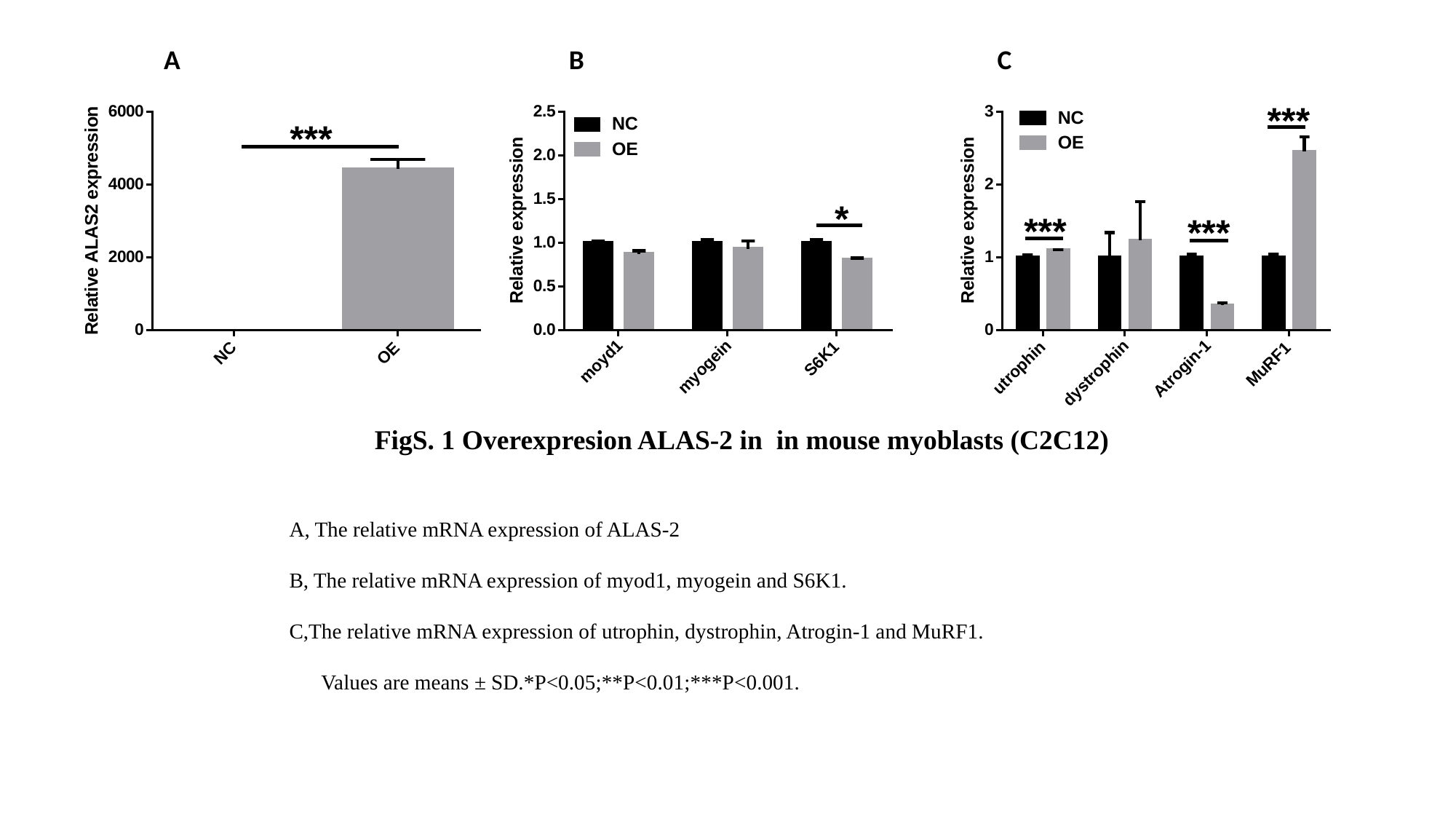

A B C
FigS. 1 Overexpresion ALAS-2 in in mouse myoblasts (C2C12)
A, The relative mRNA expression of ALAS-2
B, The relative mRNA expression of myod1, myogein and S6K1.
C,The relative mRNA expression of utrophin, dystrophin, Atrogin-1 and MuRF1.
Values are means ± SD.*P<0.05;**P<0.01;***P<0.001.
